# Tissue-Muscle Perfusion Scintigraphy of the Lower Limbs in a Patient with Type 2 Diabetes Mellitus and Peripheral Arterial Disease

**DOI:** 10.4274/mirt.73792

**Published:** 2016-02-10

**Authors:** Nevena Manevska, Daniela Pop Gjorceva, Irfan Ahmeti, Lidija Todorovska, Sinisa Stojanoski, Marina Zdraveska Kocovska

**Affiliations:** 1 University of Ss. Cyril and Methodius, Faculty of Medicine, Department of Pathophysiology and Nuclear Medicine, Skopje, Macedonia; 2 University of Ss. Cyril and Methodius, Faculty of Medicine, Department of Endocrinology, Diabetes and Metabolism, Skopje, Macedonia; 3 University of Ss. Cyril and Methodius, Faculty of Medicine, Department of Medical Physiology and Antropology, Skopje, Macedonia

**Keywords:** Perfusion, 99mTc-sestamibi, peripheral arterial disease

## Abstract

The estimation of tissue perfusion as a hemodynamic consequence of peripheral arterial disease (PAD) in diabetic patients is of great importance in the management of these patients.We present a noninvasive, functional method of ^99m^Tc-MIBI (methoxy-isobutyl-isonitrile) tissue-muscle perfusion scintigraphy (TMPS) of the lower limbs, which assesses tissue perfusion in basal conditions (“rest” study) and exercise conditions (“stress” study). Emphasis is given on perfusion reserve (PR) as an important indicator of preservation of microcirculation and its local autoregulatory mechanisms in PAD. We present a case of a 71-year-old male diabetic patient with skin ulcers of the right foot and an ankle-brachial index >1.2 (0.9-1.1). Dynamic phase TMPS of the lower limbs showed decreased and late arterial vascularization of the right calf (RC) with lower percentage of radioactivity in the 1st minute: RC 66%, left calf (LC) 84%. PR was borderline with a value of 57% for LC and decreased for RC (42%). Functional assessment of hemodynamic consequences of PAD is important in evaluating both advanced and early PAD, especially the asymptomatic form. The method used to determine the TMPS of the lower limbs, can differentiate subtle changes in microcirculation and tissue perfusion.

## Abstract (tr)

Diyabetik hastalarda mevcut periferik arter hastalığının (PAH) hemodinamik sonucu olarak gelişen doku perfüzyonunun tahmin edilmesi bu tür hastalara yaklaşımda oldukça önemlidir. Burada doku perfüzyonunu bazal (‘’dinlenme’’ çalışması) ve egzersiz (‘’stres’’ çalışması) şartlarında değerlendiren, non invaziv ve fonksiyonel bir yöntem olan ^99m^Tc-MIBI (methoxy-isobutyl-isonitrile) alt ekstremite doku-kas perfüzyon sintigrafisi sunulmaktadır. Özellikle, mikrosirkülasyonun korunması için önemli bir gösterge olan perfüzyon rezervi (PR) ve periferik arter hastalığındaki lokal otoregülatör mekanizmalara yoğunlaşılmıştır. Yetmiş bir yaşında, diyabetik, sağ ayakta cilt ülserleri olan, ayak bileği-kol indeksi >1,2 (0,9-1,1) bulunan erkek bir hasta sunulmaktadır. Alt ekstremite doku-kas perfüzyon sintigrafisinin dinamik fazında daha düşük ve sağ baldırda geç arteryel vaskülarizasyon ile birlikte 1. dakikada düşük oranda radyoaktivite saptandı, sağ baldır %66, sol baldır %84. Sol baldırda PR değeri %57, sağda ise %42 ile daha düşük bulunmuştur. PAH’ın hemodinamik sonuçlarının fonksiyonel değerlendirmesi hem ileri hem erken dönem, özellikle asemptomatik hastalıkta önemlidir. Kullanılan bu yöntem, alt ekstremite doku-kas perfüzyon sintigrafisi, mikrosirkülasyon ve doku perfüzyonundaki hafif değişiklikleri saptayabilir.

## INTRODUCTION

Peripheral arterial disease (PAD) is characterized with obstruction in lower limb circulation and impaired hemodynamics at the tissue level. The spectrum of symptoms may vary from asymptomatic forms to symptomatic disease (atypical through typical form with intermittent claudication to critical limb ischemia and gangrene, which often leads to amputation). The facts that more than half the patients remain asymptomatic, that it is associated with numerous complications and mortality are all concerning issues, which make PAD a significant health problem (1).

The screening test for PAD that is applied during clinical examination is the non-invasive ankle-brachial index (ABI) evaluation (2,3). The noninvasive, high-resolution imaging methods such as duplex ultrasound, magnetic resonance angiography and computed tomography angiography, provide anatomo-morphological information on lower extremity arteries (4,5). However, significant interest is directed to functional diagnostic procedures, which offer quantitative assessment of tissue perfusion in both basal or resting conditions (“rest” study) and exercise conditions (“stress” study) (6).

^99m^Tc-MIBI (methoxy-isobutyl-isonitrile) tissue-muscle perfusion scintigraphy (TMPS) of the lower limbs is a functional imaging method of the microcirculation detecting the hemodynamic consequences of persistent stenosis and collateral circulation development. Its application in patients with diabetes mellitus (DM), symptomatic or especially asymptomatic, is of great importance because of the specific characteristics of diabetic vasculopathy; micro- and macro-angiopathy with or without peripheral neuropathy, arterial inelasticity, and significantly low perfusion reserve of the limb (7).

## CASE REPORT

**Figure 1:**
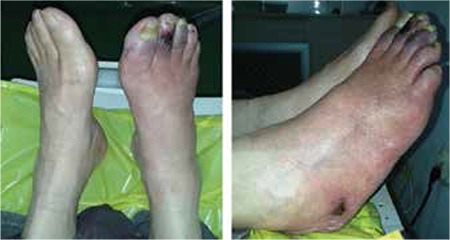
Skin ulcers on the right foot

We present a case of a 71-year-old male patient with a 7-year history of type-2 DM, as the only known risk factor for developing PAD. In the last year, he started to complain of muscle pain in the calves as well as difficulty in walking. Two weeks before presenting to the hospital, he has noticed skin changes on his right foot (Figure 1). Doppler ultrasound showed obstructive signals on both aa. dorsalis pedis as well as aa. tibialis posterior, bilaterally pulsatile aa. poplitea, and a pathologic ABI>1.2 (0.9-1.1). He was diagnosed with diabetic angioneuropathy and phlegmon of the right foot. Considering the initial skin ulcers, he was referred to nuclear medicine department where TMPS was performed, for evaluation of his tissue muscle perfusion in the lower limbs and calculation of the perfusion reserve.

### Method:

The TMPS method includes two studies: a rest study followed by a stress study. Both studies start with a dynamic phase or tissue-muscle vascularization of both calves. Then a whole body scan (WBS) is performed for tissue perfusion of the whole body. Both dynamic and planar images were performed in the posterior position.

Rest study was started with a dynamic phase after i.v application of 300 MBq of ^99m^Tc-MIBI (with a time interval of 7 minutes, 15s per frame), followed by a WBS. Dynamic phase acquisition of the stress study was started after performing 30 plantar flexions/extensions of the feet with i.v application of 600 MBq ^99m^Tc-MIBI, according to the same acquisition protocol as in the rest study. The patient was instructed to perform another 30 plantar flexions/extensions of the feet, yielding a total of 60. The WBS was then performed.

All scintigraphic studies were done with the planar technique, with two-headed gamma camera (MEDISO Nucline SPIRIT), low energy high-resolution collimator, matrix size 512x1024x16, scan speed 15 cm/min (WBS). Under these conditions, approximately 1 million impulses were collected in the rest study and around 2 million impulses in the stress study within WBS.

The data were analyzed qualitatively-visually for symmetry in vascularization (arterial, capillary and vein phase) and scored as (0) symmetric distribution, (1) mild asymmetry, (2) moderate asymmetry and (3) significant asymmetry, as well as analysis of distribution of the tracer in the regions of interest (ROI); left calf/right calf (LC/RC) (8).

Based on quantitative analyses of the dynamic phase, radioactive curves were constructed in a time manner time activity curve (TAC) above the ROI, positioned above both calves and the following parameters were determined:

*T maximum (T_max_)=Time for maximal uptake of the tracer in each calf;

*Percent of radioactivity in 1^st^ or 2^nd^ minute in calf=(radioactivity above calf in the 1^st^ or 2^nd^ minute)x100/maximum radioactivity above calf (9);

Based on quantitative analysis of the WBS and registered impulses in the ROI that was positioned over the calves, the following indices were evaluated:

*Inter-extremity index (LC/RC), for both studies;

*Calf/whole body index in %, for both studies (10);

*Perfusion reserve (PR), for both calves: as a percent of increase in the tissue blood flow in stress study in comparison with the rest study, calculated with the formula (11):

### Results:

**Figure 2:**
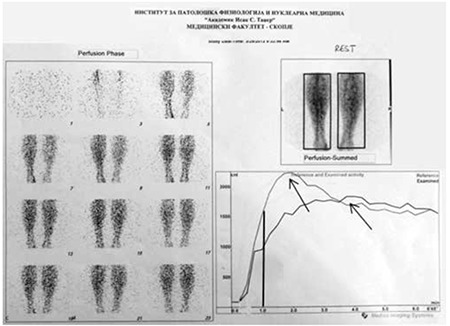
Dynamic phase TMPSLL, rest study with registered radioactivity in the 1^st^ minute (black vertical line) and peak maximum radioactivity for both calves (black arrows)

**Figure 3:**
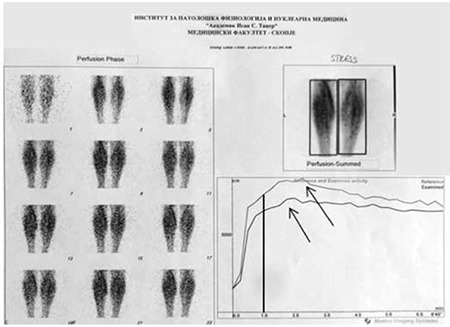
Dynamic phase TMPSLL, stress study with registered radioactivity in the 1^st^ minute (black vertical line) and peak maximum radioactivity for both calves (black arrows)

Dynamic phase of the rest study visually showed decreased and late vascularization of the RC, that was considered as a moderate asymmetry with score 2 (Figure 2). This was confirmed with prolonged T-max of the RC (210s) in comparison with that of the LC (135s), as well as with the lower percent of registered radioactivity in the 1st minute in the RC 66%, in comparison with the LC 84% (Table 1). The dynamic phase of the stress study revealed an asymmetry in the perfusion reserve both visually and on TAC. In addition, the PR in the RC was lower with lower radioactivity registered in the 1^st^ minute (5023 impulses) versus that in the LC (7461 impulses) (Figure 3, Table 1).

Inter-extremity index (LC/RC) calculated with static scintigraphy was normal in the rest study-1.05, but it was found to be pathologic for the RC in the stress study with an index of-1.14 (0.9-1.1) (Table 2).

**Figure 4:**
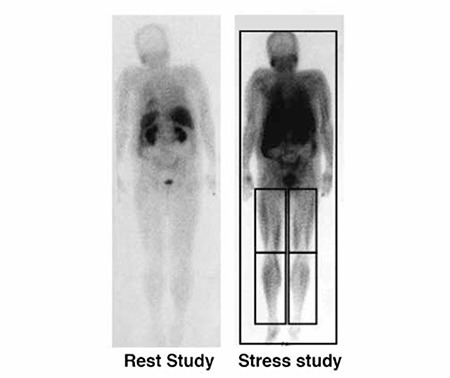
Whole body scan in both studies, posterior view with region of interest (left calf, right calf)

Perfusion reserve calculated with the above-mentioned formula on the ROI from the WBS (Figure 4) revealed a borderline value for the LC 57%, and a lower perfusion reserve for the RC 42% (reference values 50-80%) (Table 3).

The perfusion reserve was also assessed with the calf/whole body index:

LC/WBS=1.8% (rest) and 2.7% (stress) vs. RC/WBS=1.7% (rest) and 2.3% (stress).

## DISCUSSION

PAD is a recognized clinical entity since 1831. Atherosclerosis is the primary etiologic factor in the genesis of PAD. In the western world, atherosclerosis affects 10% of the population above 65 years of age, with a tendency to increase up to 22% by 2040 (12). Risk factors for its development and progression include smoking, DM, hypertension, hyperlipidemia, increased body mass index, and age (13,14).

DM is a global epidemic of the 21^st^ century. At present, there are 382 million people living with diabetes. A further 316 million with impaired glucose tolerance are at high risk for the disease-an alarming number that is set to reach 471 million by 2035 (15).

The early diagnosis of PAD is important considering the fact that PAD may remain asymptomatic in 50% of diabetic patients. Patients with both PAD and DM face serious complications, frequent hospitalizations and need for revascularization, and deteriorate quality of life. Most patients with PAD also have concomitant coronary arterial diseases (CAD), which lead to a 20% increase in the risks of nonfatal myocardial infarction and cerebrovascular stroke, as well as an increased mortality rate in 30% of such patients (16). The analysis of functional hemodynamic parameters are important for follow-up, treatment and risk stratification of patients with PAD as well as monitoring the evolution of the disease and effects of the treatment. Herein, we present a case of a patient with a 7-year history of well regulated DM and asymptomatic PAD, which probably had a long history. PAD “clinically manifested” itself within the last year with pain in the calf muscles, difficulty in walking and skin ulcers. The Doppler ultrasound and pathologic ABI value suggested the existence of PAD. It was of interest to evaluate tissue perfusion and microcirculation with macro-angiopathies, especially because of skin ulcers, where the degree of local perfusion is a predictor of healing of the wounds (17). The results of our method confirmed diabetic angiopathy in both calf regions, especially in the RC, where there was reduction in perfusion in the rest study, leading to the conclusion that local auto-regulatory tissue-muscle vascular mechanisms were being used in basal conditions. The work overload worsened the inter-extremity index as well as the perfusion reserve, confirming tissue hypoperfusion in the right along with borderline tissue perfusion in the LC. Kuśmierek et al. (10) reported the importance of this method in the evaluation of tissue muscle perfusion in 2006. They performed myocardial perfusion scintigraphy in patients with a known or newly diagnosed CAD and found significantly lower perfusion in both resting and stressed lower limb muscles in asymptomatic patients at an early phase of atherosclerosis who had normal Doppler blood flow.

Perfusion muscle scintigraphy of the lower limbs may triage patients for undergoing more invasive diagnostic methods as angiography. Soyer and Uslu (18) in 2007 presented a case report of a patient with intermittent claudication in one leg, who had normal circulation on Doppler ultrasound, markedly decreased perfusion on ^99m^Tc-MIBI muscle scintigraphy stress study, and multiple stenosis on peripheral arterial angiography.

Conservative treatment (metabolic regulation, regulation of glycemia, lipid profile, and blood pressure) is recommended for patients that present with mild or moderate obstruction. If the microcirculation is shown to be preserved by TMPS, the perfusion reserve can be accepted as a good predictor of wound healing.

## CONCLUSION

The ^99m^Tc-MIBI tissue-muscle perfusion scintigraphy of the lower limbs can differentiate subtle changes on the level of microcirculation and tissue perfusion, and can be used as a complementary method to anatomic diagnostic techniques. Functional assessment of the hemodynamic consequences of PAD is important in evaluating advanced disease as well as asymptomatic PAD, especially in assessing functional abnormalities in microvascular flow in patients with DM.

## Figures and Tables

**Table 1:**
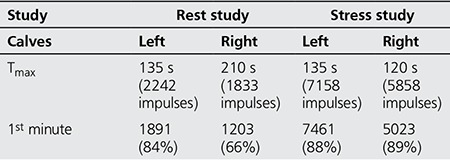
Dynamic phase of both calves in both the rest and stress studies

**Table 2:**
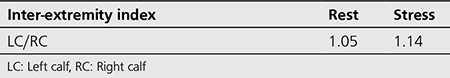
Inter-extremity index in both studies

**Table 3:**
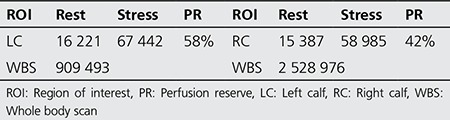
Impulses of region of interest in both studies
